# Using a human colonoid-derived monolayer to study bacteriophage translocation

**DOI:** 10.1080/19490976.2024.2331520

**Published:** 2024-03-22

**Authors:** Huu Thanh Le, Alicia Fajardo Lubian, Bethany Bowring, David van der Poorten, Jonathan Iredell, Jacob George, Carola Venturini, Golo Ahlenstiel, Scott Read

**Affiliations:** a Blacktown Clinical School, Western Sydney University, Sydney, Australia; b Storr Liver Centre, Westmead Institute for Medical Research, Sydney, Australia; c Centre for Infectious Diseases and Microbiology (CIDM), Westmead Institute for Medical Research, Sydney, Australia; d Sydney Infectious Diseases Institute, Faculty of Medicine and Health, University of Sydney, Sydney, Australia; e Department of Hepatology and Gastroenterology, Westmead Hospital, Westmead, Australia; f School of Medicine, The University of Sydney, Sydney, Australia; g Sydney School of Veterinary Science, The University of Sydney, Sydney, Australia; h Blacktown Mt Druitt Hospital, Sydney, Australia

**Keywords:** Bacteriophage, phage therapy, colonoid, intestinal permeability, translocation

## Abstract

Bacteriophages (phages) are estimated to be the most abundant microorganisms on Earth. Their presence in human blood suggests that they can translocate from non-sterile sites such as the gastrointestinal tract where they are concentrated. To examine phage translocation *ex vivo*, we adapted a primary colonoid monolayer model possessing cell diversity and architecture, and a thick layer of mucus akin to the colonic environment *in vivo*. We show that the colonoid monolayer is superior to the Caco-2 cell-line model, possessing intact and organized tight junctions and generating a physiologically relevant mucus layer. We showed, using two different phages, that translocation across the colonoid monolayer was largely absent in differentiated monolayers that express mucus, unlike Caco-2 cultures that expressed little to no mucus. By stimulating mucus production or removing mucus, we further demonstrated the importance of colonic mucus in preventing phage translocation. Finally, we used etiological drivers of gut permeability (alcohol, fat, and inflammatory cytokines) to measure their effects on phage translocation, demonstrating that all three stimuli have the capacity to amplify phage translocation. These findings suggest that phage translocation does occur *in vivo* but may be largely dependent on colonic mucus, an important insight to consider in future phage applications.

## Introduction

Bacteriophages (phages) are viruses that exclusively infect bacteria^1^, and represent the most ubiquitous, abundant and likely most diverse microorganisms on earth.^2,3^ In the human gut, and more specifically the colon where bacterial hosts are concentrated, phages are particularly abundant. Numbering up to 10^10^ particles/g of dry stool, phages play an essential role in controlling and maintaining a stable healthy microbiome.^4,5^ In addition to phages, microbial dynamics within the gut are affected by gastrointestinal immune responses including mucus, IgA, and antimicrobial peptide production, highlighting the trans–kingdom interactions that ultimately define the microbiota.

Although gut microbes including phages are primarily located within the gut lumen, translocation of viable bacteria or bacterial components such as bacterial lipopolysaccharide from the gut into systemic circulation can occur, even in the absence of disease.^6–9^ Evidence suggests that gut phages can translocate across the intact gut epithelium based on the identification of phage DNA in lymph node, blood, urinary tract and even sterile sites such as cerebrospinal fluid.^10,11^ The presence of viable phages at these sites, even in seemingly healthy subjects, has raised interest in the mechanisms of phage translocation and their contribution to disease. Rapid translocation of phages *ex vivo* across continuous cell lines, such as Caco-2 colon carcinoma cells, has been observed,^12,13^ leading to speculation that billions of phages could translocate every day across the human gut.^12^ These findings can potentially change the way we view interactions with phages and the mammalian body, but must be interpreted with caution as cell lines often lack physiological protein expression and cell diversity relevant to *in vivo* systems. The Caco-2 cell line, for example, is deficient in mucus secretion and does not possess the multitude of intestinal cell types present in the human colon.^14^ Further research is necessary to establish the relationship between the gut mucosa and phage translocation, preferably using more relevant and more sophisticated *ex vivo* models.

An increase in bacterial translocation has been associated with many chronic gastrointestinal and hepatic diseases such as liver disease (ALD), metabolic-associated fatty liver disease (MAFLD) and inflammatory bowel disease (IBD).^15–18^ This likely occurs due to the loss of gut barrier integrity leading to an increase in intestinal permeability, a phenomenon called “leaky gut”.^18,19^ Among the barriers likely to be disrupted are the mucus layer and cellular tight junctions that are crucial for gut epithelial integrity and commonly found to be impaired in disease conditions.^18–20^ The effect of such impaired epithelial integrity on phage translocation has not yet been studied, but defining these effects is particularly important in the evolving era of phage therapy, where orally administered phage preparations are being considered to treat multidrug resistant (MDR) bacterial infections.^21–28^ Indeed, we must determine whether high titer, orally administered phages can translocate across the gastrointestinal epithelium in humans and enter circulation, as some phages have been shown to be highly immunogenic.^29–31^

In this study, we used an *ex vivo* organoid-derived monolayer model generated from human colon biopsies to investigate phage translocation. The roles of mucus and tight junction proteins in phage translocation were also studied using both this colon-derived model and the Caco-2 cell-line model for comparison. In addition, phage translocation in gastrointestinal and hepatic diseases was examined *in vitro* using both intestinal epithelial models stimulated with various insults to mimic disease states.

## Methods

### Phage production and quantitation

Phage and bacterial strains are listed in Table 1.

**Table 1.** Phage characteristics.

Phage	Morpho- logy	Genome material	Phage size (width and length)	Bacterial host	Phage titre (PFU/ml)	Endotoxin level (Unit//ml media)	GenBank Accession no./Sequence
Ec70	Myovirus	163 Kbp DNA	87.505 nm 239.654 nm	MDR *E. coli* JIE 3454	10^11^-10^14^	1.8–3.9 x 10^−4^	SRA accession: PRJNA764821; SAMN40258554
Kp127	Siphovirus	113.7 Kbp DNA	82.966 nm 374.799 nm	MDR *K. pneumonia* ATCC 13,883	10^10^-10^13^	2.3–5.5 x 10^−4^	MN434096

Phages were amplified in 50 ml of bacterial culture (OD_600_ 0.3) for 3 hours. Phage filtrates were purified by PEG-precipitation following established protocols.^32,33^ PEG was removed by adding 1 M KCl and incubating for 20 minutes at 4°C before centrifuging at 4,700 RCF at 4°C for 10 minutes. Endotoxin was removed from the phage suspension using cesium chloride (CsCl) gradient ultracentrifugation, as described previously.^32^ Endotoxin concentrations in the final phage products were measured using the Pierce™ Limulus amebocyte lysate (LAL) Chromogenic Endotoxin Quantitation Kit (Thermofisher, #88282), following the manufacturer’s instructions. Phage titers (PFU/ml) were measured using the double agar overlay method and spot testing as we have done before.^33^

### Tissue collection

Colon biopsies were obtained from 20 consented adults during routine colonoscopies at Westmead, Blacktown Mount-Druitt Hospitals. A total of 6 to 8 biopsies were collected in a sample jar using endoscopic biopsy forceps and stored in cold DBPS for a maximum of 3 hours before being processed. Project ethics (HREC Reference Number: (4192) AU RED HREC/15 WMEAD/11) were approved by Western Sydney Local Health District Human Research Ethics Committee, NSW, Australia.

### Colonoid culture

Biopsies were transferred into 15 ml tubes and washed three times with 10 ml of cold DBPS. Crypts were isolated by incubating with chelating buffer containing 2.5 mM of EDTA, 1× penicillin–streptomycin (Sigma-Aldrich, #P4333), and 10 µg/ml gentamicin (Sigma-Aldrich, #G1264), at 37°C, with gentle shaking for 30 minutes. Biopsies were then carefully washed with 10 ml of cold DBPS to remove any remaining debris, followed by gentle shaking 20–30 times in 5 ml of cold DBPS to release crypt structures from the submucosal tissue. Biopsy debris was removed using a P1000 pipette, and crypts were pelleted by centrifuging at 50 RCF, 4°C for 3 minutes. Crypts were resuspended in 1 ml of 10% FBS in cold DBPS, transferred to a 1.5 ml Eppendorf tube, and centrifuged at 150 RCF at 4°C for 3 min. After removing as much supernatant as possible, 40 µl of cold Matrigel was added, and crypts were quickly resuspended by pipetting and plated in two wells of a 24-well plate (20 µl/well). The plate was placed upside-down in the cell culture incubator for 45 minutes to allow Matrigel to solidify, after which 500 µl of expansion media (Advanced DMEM containing 10 µg/ml gentamicin) was added to eliminate possible pen/strep-resistant bacteria. Within 24 hours, crypts became circularized as colonoids, and every 2–3 days, expansion media without gentamicin was changed.

### Colonoid monolayer development

6.5 mM transwell® inserts with 0.4 µm pore polyester membranes were equilibrated in 24-well plates at 37°C for 15 minutes. Inserts were washed with 200 µl of warm DBPS, and 100 µl of coating material (i.e., Matrigel at 200, 40, 20 µg/ml, or collagen 1 at 100-0.2-0.1-20 µg/ml) diluted in cold DBPS was added onto each insert. The inserts were then incubated in a 37°C incubator for at least 2 hours and used within 24 hours. Alternatively, the plate was sealed with parafilm and stored at 4°C for later use. Before use, excessive DBPS was carefully removed from the insert by pipetting, and inserts were gently washed with 100 µl warm DBPS.

Colonoids cultured in expansion media for 4 days were used to develop colonoid monolayers. Colonoids were isolated from Matrigel using cold DBPS as described in section 2.2.1. Fifty µl/well of TrypLETM was added and incubated in a 37°C water bath for 3 minutes. Ten milliliters of cold 10% FBS in DBPS was immediately added, and colonoids were pelleted by centrifuging at 100 RCF, 4°C for 3 minutes. After removing most of the supernatant, leaving around 200 µl, colonoids were fragmented into single-cell suspensions by vigorously pipetting with 200 µl tips 40 times. One milliliter of 10% FBS in DBPS was added and filtered through a 40 µm cell strainer into a 1.5 ml Eppendorf tube. Cells were then pelleted by centrifuging at 250 RCF, 4°C for 3 minutes. After removing the supernatant, 100 µl of expansion media/well was added to resuspend cells. One hundred µl of cell suspension was seeded on equilibrated transwell inserts. Six hundred µl of expansion media was added to the basolateral chamber. The transwell plate was then put back in the cell culture incubator to allow cell adherence for 48 hours. Monolayer confluence was observed after 10–14 days of culture. To differentiate monolayers, differentiation media #1 was added to cells for at least 3 days before future work.

### Caco-2 monolayer development

Caco-2 cells were cultured in 24-well plates until reaching confluency. Cells were detached by treating with 200 µl TrypLETM/well for 3 minutes and then resuspended in the culture media. Cells were dissociated by vigorous pipetting and collected by passing through a 40 µm cell strainer. A total of 5 × 10^5 cells were seeded on collagen I-coated transwell inserts with 100 µl of media in the insert and 600 µl of media in the basolateral chamber. The cells were allowed to grow until reaching confluency, which typically took 3 days. The Caco-2 monolayer was cultured for either 5 days or 21 days with media changes every 3 days.

### Measurement of Transepithelial/trans-endothelial electrical resistance (TEER)

TEER of monolayer models was measured using the EVOM™3 with STX4 electrodes on resistance mode with resistance range of 10,000 ohm. TEER measurement was conducted by placing the anode (short electrode) in the insert and cathode (long electrode) in the basolateral chamber and obtaining a stable reading within 1 minute. The monolayer TEER reading was subtracted by the reading on blank collagen I coated transwell. The final TEER of the monolayer was calculated as TEER (ohms.cm^2^ = TEER reading (ohms) x (0.33 cm^2^)

### Measurement of FITC-dextran influx

Media was removed from insert and basolateral chamber of transwell and replaced with 100 µl of warm HBSS with 100 µg/ml of FITC-dextran 4kDa and incubated in 2 hours. A series of FITC-dextran standards (STD) was created from 10 µg/ml to 0.78125 µg/ml by serial dilution with HBSS. After 2 hours, 100ul of basolateral HBSS or STDs was used to measure fluorescence intensity at excitement and emission wavelength of 485 nm and 550 nm respectively. The concentration of diffused FITC-dextran was calculated using the standard curve.

### Phage translocation assay

To examine the mucus layer and cell junctions’ impact on phage translocation, three different treatments were conducted on both 21-day cultured Caco-2 and differentiated colonoid monolayers. Firstly, the differentiated monolayer model was cultured in a semi-wet condition with 20 µl of media in the insert for 3 days to stimulate mucus secretion [39]. To test the removal of mucus, 100 µl of 10 mM N-acetyl cysteine in warm DBPS was added to the apical chamber and incubated for 30 minutes with gentle shaking every 10 minutes. The dissolved mucus was removed, and the insert was washed with 100 µl of warm DBPS before adding 100 µl of differentiation media.

To examine tight junction disruption, the media in the apical chamber was washed with warm DBPS and replaced with 100 µl of 2 mM EDTA in DBPS.^34^ The media in the basolateral chamber was also replaced with warm DBPS. The monolayer was incubated for 10 minutes in a 37°C incubator before differentiation media was put back in both the apical and basolateral chambers. Mucus-depleted and tight junction-disrupted models were created on the same day as the phage translocation assay. TEER was also measured before phage treatment to examine monolayer integrity.

To create disease models, monolayers were cultured in their respective media supplemented with either 0.2% ethanol, 0.3 mM (2 palmitic acid (PA): 1 oleic acid (OA)) or a combination of 10 µg/ml IL-1β, 10 µg/ml IL-6 and 10 µg/ml TNF-α for 24 hours. These treatments (applied to both the apical and basolateral chambers) have been previous used to stimulate alcoholic lover disease (ALD),^35^ Metabolic associated fatty liver disease (MAFLD)^36^ and inflammation bowel disease (IBD).^37–39^

To examine phage translocation, a mix of 10^7^ Ec70 and 10^7^ Kp127 was added to the insert. After 2 hours, basolateral chamber media was collected and replaced with new media. After 24 hours, basolateral chamber media was collected again.

### Monolayer fixing and histology staining

The media was drained from the insert by inverting it on absorbent paper, and the basolateral chamber was aspirated. One hundred microliters and 600 µl of −20°C methacarn were added to the insert and basolateral chamber, respectively, and incubated for 15 minutes to fix the cell and mucus layers. The fixative was then removed and exchanged with 30% sucrose overnight at 4°C. The polycarbonate membrane was removed with a scalpel and cut in halves. One-half of the membrane was embedded with OCT in a cryomold on dry ice. The sample block frozen in OCT was sectioned at 5 µm thickness using the Cryostat N×70(ThermoFisher) and adhered to SuperfrostTM Plus slides (ThermoFisher, #12-550-15). Slides were dried for 10 minutes and stored at −20°C.

Before staining, sections were post-fixed in 10% Neutral Buffered Formalin for 10 minutes and then gently washed in distilled water to remove OCT. For hematoxylin and eosin (H&E) staining, sections were first stained with modified Mayer’s hematoxylin for 5 minutes, followed by washing with distilled water, then enhanced with bluing solution with Scott’s Blue reagent for 1 minute. Slides were washed and exchanged in 100% ethanol for 1 minute before counter-staining with eosin for 45 seconds. For Alcian blue staining, sections were flushed with 3% acetic acid for 3 minutes, then stained with 1% Alcian Blue in 1% acetic acid for 30 minutes. The slides were then washed in distilled water and counter-stained with Nuclear Fast Red solution for 10 minutes. For PAS staining, the section was oxidized with 0.5% periodic acid solution for 5 minutes, then stained with Schiff reagent for 15 minutes. The slide was washed in distilled water before counterstaining with Mayer’s hematoxylin for 1 minute. After staining, sections were dehydrated once in 70% ethanol and twice in 100% ethanol (1 minute each), cleared three times with xylene (2 minutes each), and a coverslip was added with mounting media. Sections were visualized using the Olympus LS microscope equipped with an LC35 camera (Olympus).

### Immunofluorescent staining and imaging

The remaining half of cell monolayers on transwell membranes was cut in half, and a quarter of the membrane was blocked with 3% goat serum in DBPS for 1 hour. A combination of primary antibodies, mouse anti-human Muc2 and rabbit anti-human ZO1, or mouse anti-human ChgA and rabbit anti-human Lyz (all at a 1:500 dilution from stock), was added with 1% goat serum and incubated overnight. Samples were washed three times in DBPS (5 minutes each) before secondary fluorescent-tagged antibodies at a 1:2000 dilution in DBPS (Alexa 488 anti-mouse Ab and Alexa 594 anti-rabbit Ab) were added and incubated for 2 hours. Samples were washed three times with DBPS as before, and nuclei were counterstained with Hoechst 33,342 (1:4000 dilution in DBPS) for 10 minutes. The samples were washed once more with DBPS and cover slipped with ProLong™ Gold Antifade Mount (ThermoFisher Scientific, # P10144). Cell monolayers were imaged with the confocal microscope Leica TCS SP5 with Z-stacking at a thickness of 0.2 µm. Images were processed with Fiji (ImageJ).

### Data availability

The authors confirm that the data supporting the findings of this study are available within the article [and/or] its supplementary materials. Ec70 and Kp127 phage genome sequences are accessible via https://www.ncbi.nlm.nih.gov/genbank/.

## Results

### Development of a colon-derived organoid monolayer model to study phage translocation

**Figure 1.**
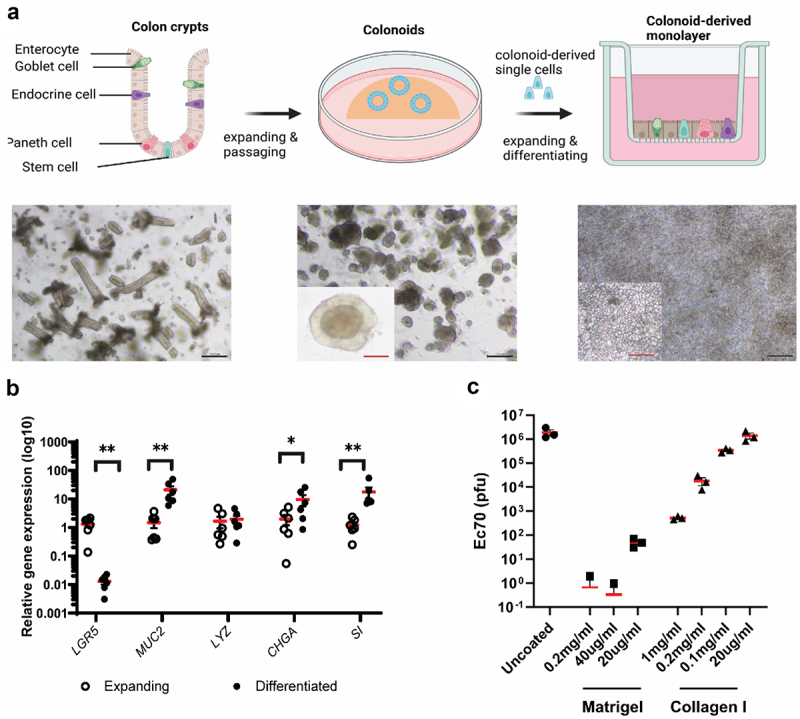
Development of colon-derived epithelial monolayer for phage translocation study.

To generate a physiologically relevant model of the human colon, we adapted a colon-derived monolayer model to study phage translocation *ex vivo* .^40^ Human colon-derived organoids, termed colonoids, were cultured in Matrigel using established methodologies^41^ (Figure 1a). Expanded colonoids underwent dissociation using TrypLE and were subsequently seeded onto 0.4 µM pore transwell inserts coated with Matrigel to enable the development of a monolayer comprising undifferentiated colon epithelial cells. Assessment of three differentiation media^41–43^ was conducted to identify the formulation that best stimulated the expression profile of differentiated intestinal epithelial cell markers (mucin 2 [MUC2] for goblet cells, lysozyme [LYZ] for Paneth cells, chromogranin A [CHGA] for endocrine cells, and sucrose isomaltase [SI] for enterocytes) in colonoids, as compared to isolated colon crypts (Supplementary Table S1; Supplementary Figure S1). In addition to a reduction in stem cell marker leucine-rich repeat-containing receptor 5 (LGR5) expression levels and an induction of enterocyte (SI) and enteroendocrine (CHGA) markers, differentiation media #1 (Supplementary Table S1) elicited the highest expression of MUC2 in the colon-derived monolayer and was chosen for subsequent differentiation of colonoid monolayers, given the crucial role of mucus in maintaining intestinal barrier integrity and in serving as a niche for phages.^44,45^ The differentiation status of the colonoid monolayer in Formula #1 compared to undifferentiated cell monolayers is shown in Figure 1b.

a. Colon crypts (left) were isolated from colon biopsies and grown into 3D colonoids (centre) which were then dissociated into single cells and seeded on transwells to form a colon-derived epithelial monolayer (right). b. Expression of colon epithelial cell markers in expanding and differentiated layers. Markers: *LRG5*, stem cells; *MUC2*, Goblet cells; *LYZ*, Paneth cells; *CHGA*, endocrine cells; *SI*, enterocytes. Wilcoxon test, *n*=6, * *p* < .05, ** *p* < .01. c. Optimising transwell-coating substances to allow free diffusion of translocated phages. Black bar scale,200 nm; red bar scale, 50 nm.

To ensure that the coating matrix did not impede the diffusion of translocated phages across the epithelium, we applied the two most common culture matrix substances, namely Matrigel forming a complex and dense matrix, and collagen-I creating a uniform and sparse net, onto the transwell inserts at varying concentrations. Myovirus vB_EcoM-JIPh_Ec70 (Ec70; SRA accession: PRJNA764821; SAMN40258554) (10^7^ plaque-forming units or PFU) was then introduced into the coated transwell inserts. The rate of translocation was determined by measuring phage titer in the basolateral chamber after a 3-hour incubation period. Comparative analysis against uncoated transwells revealed that Matrigel coating, even at concentrations as low as 20 µg/ml, significantly impeded the diffusion of the majority of phages (99.99% reduction; Figure 1c). Conversely, collagen-I coating allowed a greater translocation of phages across the epithelium (Figure 1c). Notably, collagen I at a concentration of 20 µg/ml demonstrated a diffusion profile similar to that of uncoated transwells, thus warranting its selection for the development of the colonoid monolayer. Importantly, this concentration of collagen was sufficient for transwell cell adherence and monolayer formation.

**Figure 2.**
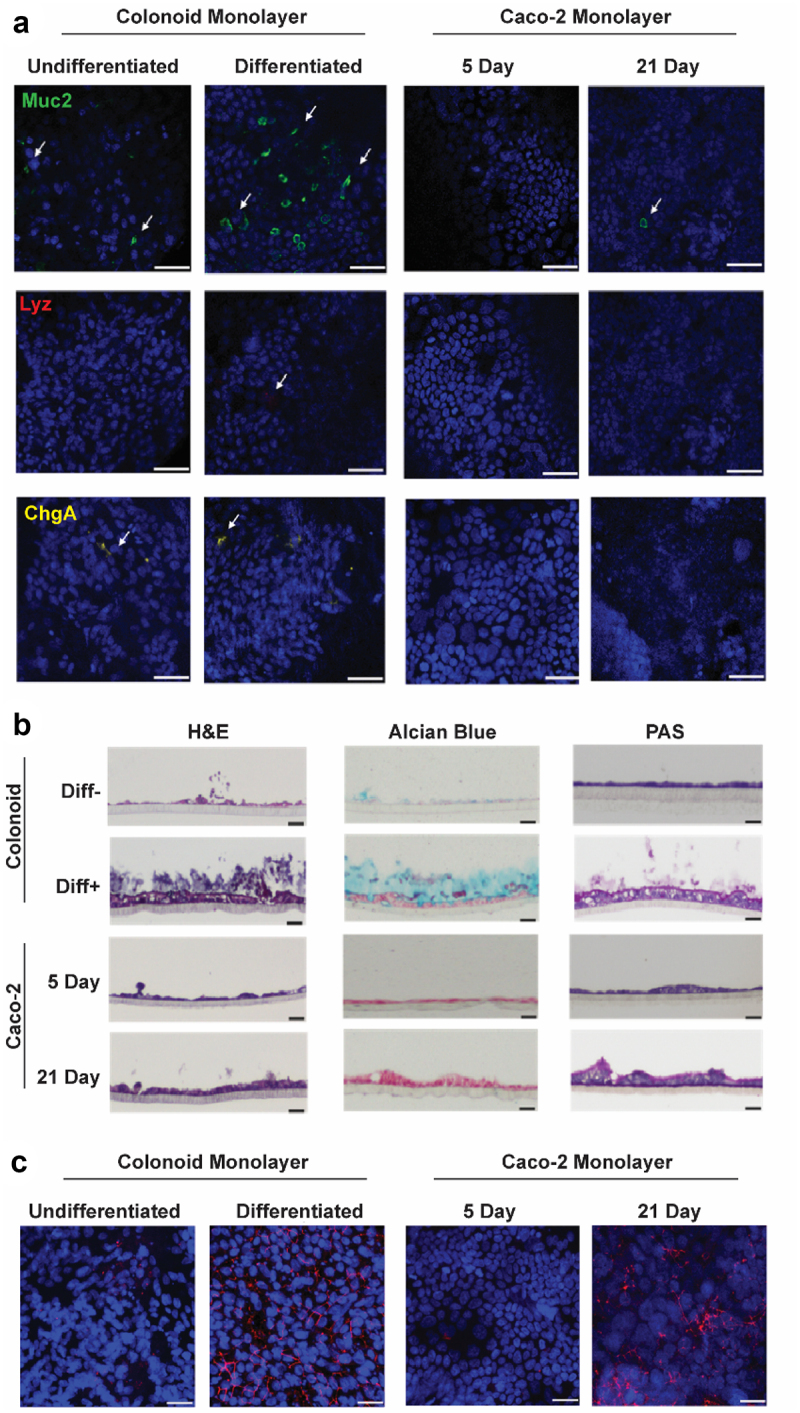
Characterisation of colon epithelial monolayer models.

To serve as a comparison for phage translocation, a Caco-2 monolayer was grown to confluency for 5 days as described previously,^12^ and then additionally cultured for 21 days, at which point cells become further differentiated, cell junctions organize, and microvilli develop.^46^ The differentiation status of Caco-2 and colonoid monolayers were assessed by immunofluorescent labeling of MUC2, LYZ and CHGA (Figure 2a). All three markers were detected in differentiated colonoid monolayers. MUC2 (goblet cells) and CHGA (enteroendocrine cells) were also identified in the undifferentiated colonoid monolayer but to a lesser degree. In contrast, markers indicative of mature intestinal cells were notably absent in Caco-2 cells cultured for 5 days. Surprisingly, a small number of cells expressing MUC2 were observed in Caco-2 cells cultured for 21 days, contrary to reports showing the absence of secreted mucins in Caco-2 cells.^14,47^

a. Immunofluorescent staining for MUC2, LYZ and CHGA markers for goblet cells, Paneth cells and endocrine cells, respectively, on differentiated (Diff+) and undifferentiated (Diff-) colonoid-derived monolayers and Caco-2 monolayer cultured in 21 days (21 Day) and 5 days (5 Day). b. Haematoxylin and eosin (H&E), Alcian blue and *Periodic acid–Schiff* (PAS) staining on vertical section of the monolayer models. c. Immunofluorescent staining for Zonula Occludin(ZO-1) on the monolayer models. Scale bar = 20 µm.

Histological staining using Hematoxylin and Eosin (H&E), Alcian blue, and Periodic acid – Schiff (PAS) was also performed to examine cell morphologies and mucus production. H&E staining (Figure 2b) confirmed the presence of intact cell monolayers in all four models. Caco-2 cells cultured for 21 days and differentiated colonoid cells exhibited a columnar morphology, while Caco-2 cells cultured for 5 days and undifferentiated colonoid cells displayed a squamous appearance. Alcian blue and PAS staining, targeting acidic and neutral mucins, respectively, revealed a substantial layer of mucus atop the differentiated colonoid monolayer, with evidence of detached cells within the mucus layer. A thinner layer of mucus was observed on the undifferentiated colonoid monolayer. In contrast, only PAS staining (neutral mucins) was detected on the 21-day Caco-2 monolayer, but not on the 5-day. Immunofluorescent labeling of Zonula Occludin (ZO-1) highlighted a network of tight junctions connecting cells in the differentiated colonoid monolayer and the 21-day Caco-2 cells (Figure 2c). ZO-1 was detected in the undifferentiated models but with lower and disorganized expression.

**Figure 3.**
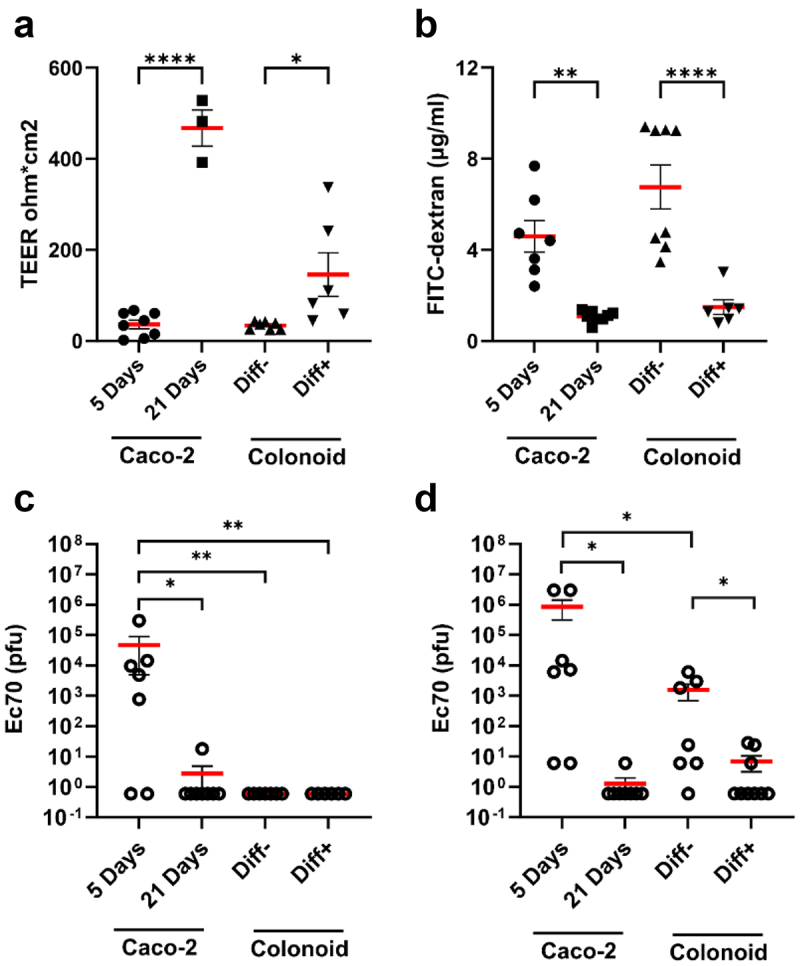
Permeability of colon epithelial models and phage translocation across these models.

Monolayer integrity was next assessed using two methods: Transepithelial Electrical Resistance (TEER) and FITC-dextran influx (Figure 3a, b). Notably, Caco-2 cells cultured for 5 days and undifferentiated colonoid monolayers exhibited significantly lower TEER values than Caco-2 cells cultured for 21 days and differentiated colonoid monolayers, respectively. Similarly, the permeation of FITC-dextran was markedly higher in 5-day Caco-2 monolayers and expanding colonoid monolayers compared to the 21-day Caco-2 and differentiated colonoid monolayers, respectively.

a. Trans-epithelial electrical resistance (TEER) was measured before phage treatment showing increased resistance in differentiated/mature monolayers. b. Following the phage translocation assay, FITC-dextran was used to measure the degree of permeability of cell monolayers by measuring FITC fluorescence intensity in the basolateral chamber media. c, d. Ec70 phage titre collected from the basolateral chamber at 2 h (c) and 24 h (d) after administration of EC70 (10^7^ PFU) to the transwell. Unpaired t-test, * *p*< .5, ** *p*< .01, ***p< .005. Diff-, undifferentiated, Diff+, differentiated.

To investigate phage translocation, we used Ec70, an *Escherichia coli* myovirus (SRA accession: PRJNA764821; SAMN40258554). Media from Caco-2 and colonoid monolayers was replaced with fresh media containing 10^7^ Ec70 PFU and translocation was quantified at 2 h (Figure 3c) and 24 h (Figure 3d). After 2 hours incubation, phages were detected in the basolateral chamber of 5-day Caco-2 monolayers exclusively, with an average quantity of 10^4^ PFU present. The number of translocated phages increased to 10^6^ PFU after 24 hours. In undifferentiated colonoid monolayers, an average of 10^3^ phages translocated after 24 hours. In contrast, minimal phage translocation (<10 PFU) was observed in the 21-day Caco-2 and differentiated colonoid monolayers. These data support the roles of mucus and tight junctions (Figure 2) as critical barriers for phage movement across intestinal epithelial models.

### Intact mucus layer and cell junctions prevent phage translocation in healthy intestinal epithelium

Phages have been shown to concentrate in the mucus layer.^4,44,45^ While mucus is a natural barrier against luminal microbes, the proximal location of mucus-bound phages to the epithelial layer in the gut has been suggested to facilitate phage translocation.^44^ To investigate whether mucus can enhance or restrict phage translocation, differentiated colonoid monolayers were cultured in different conditions to increase or decrease the amount of adherent mucus. To stimulate mucus secretion, colonoids were cultured in semi-wet conditions as previously performed by Navabi et al..^48^ Alternatively, mucus was removed by incubation with N-acetylcysteine, which disrupts disulfide linkages between mucin molecules, followed by extensive washing.^49^ Finally, monolayers were also treated with EDTA to deplete mucus and disrupt cell junctions as a positive control for phage translocation, as was done before.^34^ The same treatments were applied to 21 day Caco-2 monolayers for comparison.

**Figure 4.**
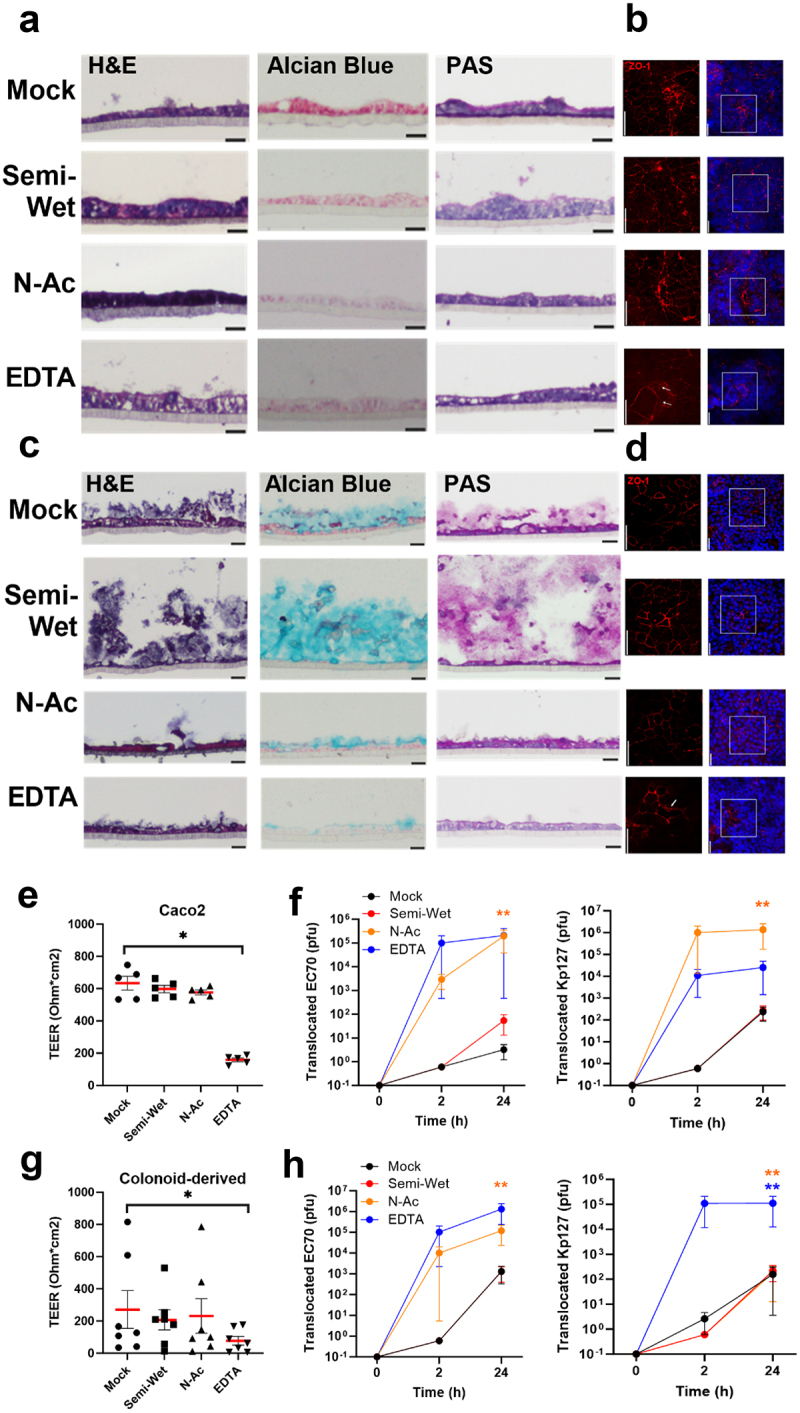
Monolayer integrity and phage translocation following mucus stimulation and depletion.

H&E staining showed an intact layer of cells on both Caco-2 and colonoid monolayers pre-treatment conditions (Figure 4). Alcian blue staining showed no mucus production in Caco-2 monolayers, even in semi-wet conditions. By contrast, colonoid monolayers showed thicker mucus (~100 µm) when cultured in semi-wet conditions. Conversely, both N-acetylcysteine and EDTA removed a significant amount, but not all, the mucus on colonoid monolayers. Immunofluorescent labeling of ZO-1 showed intact tight junctions surrounding epithelial cells in Caco-2 and colonoid monolayers in all culture conditions except EDTA treatment, where a disrupted tight junction architecture was observed.

Histological staining and immunofluorescent labelling of ZO-1 in Caco-2 monolayers (a, b) and colonoid monolayers (c, d) to assess monolayer composition, mucus production and tight junction expression. Trans-epithelial electrical resistance (TEER) was measured in all treatments, with only EDTA causing a significant reduction in both models (e, g). Phages Ec70 and Kp127 were added to Caco-2 (f) and colonoid monolayers (h), and phage translocation was quantified following stimulation of mucus production (semi-wet), mucus depletion (N-Ac, N-acetylcysteine treatment) and EDTA treatment. Mann-Whitney *U* test, * *p*< .5, ** *p*< .01.

Phage translocation was next examined following mucus stimulation or depletion. TEER measured on Caco-2 and colonoid monolayers showed a significant reduction only following treatment with EDTA (Figure 4), consistent with previous evidence.^50^ To assess the role of mucus in the translocation of different phage types, we applied 10^7^ PFU Ec70 (240 nm myovirus) or JIPh_Kp127 (Kp127; 375 nm siphovirus; Genbank MN434096.1)^33^ phages to cell monolayers. After 2 hours and 24 hours incubation, basolateral media was collected and phage titer quantified. Following 2 hours incubation, only mucus depleted and EDTA treated Caco-2 and colonoid monolayers allowed translocation of both phages. At 24 hours, phage translocation could be quantified in all treatments in both models, with a significant increase following mucus removal and EDTA treatment (*p* <  .001-p < .05 following mucus depletion, *p* < .05-N.S. following EDTA). Phage translocation remained minimal (10^1^-10^2^ PFU) in control and semi-wet culture treatments.

### Assessing phage translocation following treatment with aetiological drivers of intestinal permeability

**Figure 5.**
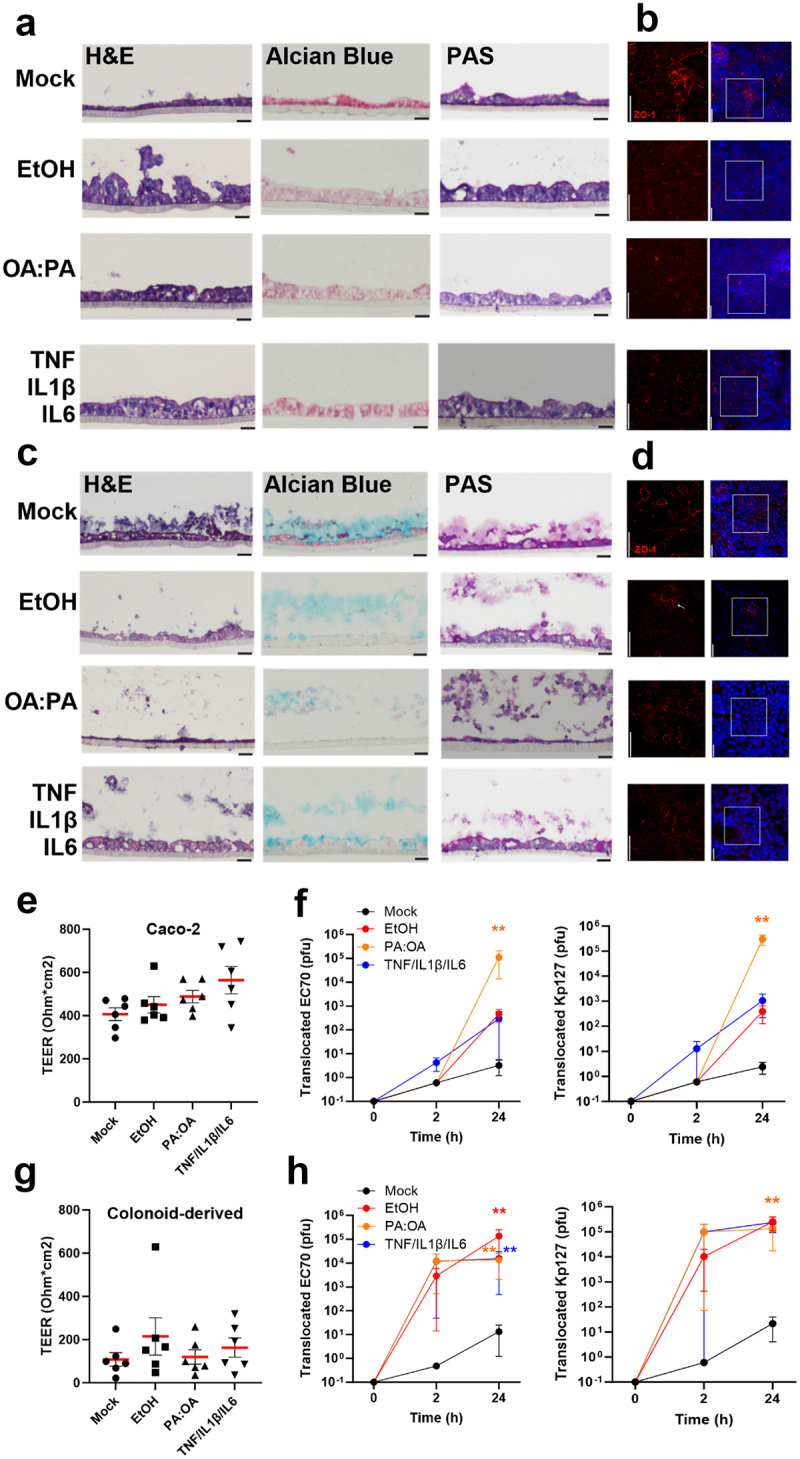
Monolayer integrity and phage translocation following stimulation of intestinal permeability with alcohol, lipid and cytokines.

Bacterial translocation and gut permeability are well described among gastrointestinal diseases such as ALD, MAFLD, IBD.^15,16,51^ To examine phage translocation in monolayer models mimicking these disease states, we treated Caco-2 and colonoid monolayers with either 40 mM ethanol, 0.25 mM of 2:1 palmitic:oleic acid or a cocktail of IL-1β, IL-6 and TNF-α (10 ng/ml each) for 24 hours to model ALD, MALFD, and IBD, respectively (Figure 5). Treated Caco-2 monolayers became thickened (Figure 5a), whereas colonoid monolayers were largely unchanged (Figure 5c), except following fatty acid treatment which resulted in thinner, more squamous-shaped cells. As before, Alcian blue staining was largely absent in Caco-2 monolayers, whereas treated colonoid monolayers demonstrated a detachment of the mucus layer accompanied by a reduction in staining intensity. PAS staining appeared to be reduced only following fatty acid treatment in both models.

Histological staining and immunofluorescent labelling of ZO-1 in Caco-2 monolayers (a, b) and colonoid monolayers (c, d) following stimulation for 24 hours with aetiological drivers of intestinal permeability including 0.2% ethanol (EtOH), 0.3mM 2 oleic: 1 palmitic acid (OA:PA) and inflammatory cytokines (10 µg/ml each TNF, IL1β, IL6). TEER was measured in all treatments, indicating no significant change (e, g). Phages Ec70 and Kp127 were added to Caco-2 (f) and colonoid monolayers (h), with phage translocation measured after 2 and 24 hours. Mann-Whitney *U* test, * *p*< .5, ** *p*< .01.

Immunofluorescent targeting of ZO-1 showed reduction and disrupted tight junctions in all disease-mimicking conditions compared to the untreated controls in the Caco-2 model (Figure 5b). Similar observations were made for alcohol treated colonoid monolayers (Figure 5d), showing the dissociation of tight junctions (Figure S5.23). Colon-derived monolayers treated with fatty acids or cytokines also showed a reduction in ZO-1 signal compared to the untreated control. Treating Caco-2 monolayers with ethanol, fatty acids or cytokines caused a noticeable but non-significant increase in TEER (Figure 5e), perhaps reflecting the thicker epithelium observed in treated monolayers. No significant difference in colonoid monolayer TEER was measured (Figure 5g).

To test phage translocation, a mix of Ec70 and Kp127 (10^7^ PFU each) was added to treated Caco-2 and colonoid monolayers. Basolateral media was collected at 2 and 24 hours and phage titer quantified. Surprisingly, phage translocation across Caco-2 monolayers was largely absent after 2 hours (Figure 5f) but was observed in all treated colonoid monolayers (Figure 5h, *p* > 0.05). At 24 hours, translocation of both Ec70 and Kp127 across Caco-2 monolayers increased in all treatments, reaching significance following only fatty acid exposure (*p* < .01). Ec70 phage translocation was significantly increased in colonoid monolayers in all treatments after 24 hours, whereas Kp127 only increased significantly in response to fatty acid treatment. Interestingly, both ethanol and cytokine treatment stimulated phage translocation in colonoid monolayers (10^4^-10^5^ PFU) as compared to Caco-2 monolayers (10^3^ PFU).

## Discussion

The human colon, as part of the gastrointestinal tract, contains the largest and most diverse microbiome in the human body, and thus the highest concentration of phages.^52,53^ Detection of gastrointestinal phages in “sterile” sites of the body, such as the blood or cerebrospinal fluid, suggests that they can be translocated from their primary gut niche. Indeed, oral administration of high-titer phages in mouse studies has shown translocation into blood and tissue,^54^ even in the absence of disease.^55^ As phages have the potential to elicit context-dependent inflammatory, and anti-inflammatory responses in systemic circulation,^29–31,56^ physiologically relevant models to understand gastrointestinal phage translocation are sorely needed. To study phage translocation in the colon and define factors that enhance and prevent their migration, we have generated an *ex vivo* colon-derived monolayer model, based on previous works by In et al..^40^ Using two different phages, we have demonstrated that phage translocation in this model is rare, but significantly amplified when mucus is depleted or following insult with factors that drive intestinal permeability, like alcohol, fat, and inflammatory stimuli.

By measuring transcriptional and protein markers for enterocytes, goblet cells, Paneth cells and enteroendocrine cells, we confirmed the presence of diverse cell types in the colonoid monolayer, representative of the diversity of the human colon. In addition, we showed that our colonoid monolayer model possesses a thick mucus layer absent in the Caco-2 immortalized cell line. As phages adhere to colonic mucus *in vivo* ,^45^ we hypothesized that the absence of sufficient mucus production in immortalized cells like Caco-2 would artificially facilitate phage translocation in experimental set-ups. In our model, we also optimized the transwell extracellular matrix (ECM) coating to ensure it does not interfere with phage translation. Matrigel basement membrane matrix produced by mouse sarcoma cells was found to obstruct phage diffusion, while collagen I at 20 µg/ml allowed phages to freely diffuse. These data highlight the multiple physiological barriers that must be bypassed for gastrointestinal phage to enter systemic circulation in a healthy gut: mucus, colonic epithelium, ECM, and finally the capillary endothelium. In addition to acting as a physical barrier, mucosal ECM is made up of numerous components that can directly bind phage including fibronectin, heparin, and gelatin that have the potential to hinder further dissemination.^57^ Phages that translocate across the colonic epithelium may also encounter resident mucosal phagocytes including macrophages and dendritic cells,^58^ presenting yet another obstacle that must be surpassed before reaching the bloodstream. Movement across enteric capillary beds is perhaps the easiest step, as capillary walls are one cell thick, and are more likely to resemble the cell monolayers that were recently shown to enable significant phage translocation via transcytosis.^12,13^

Experimental permeability tests and histological staining demonstrated distinct characteristics of colonoid monolayers that highlight their superiority as a model to test translocation. Specifically, differentiated colon-derived monolayers present a thick layer of mucus, organized cellular ZO-1 expression, and improved barrier integrity compared to undifferentiated colonoid monolayers and 5-day Caco-2 monolayers. In comparison, 21-day Caco-2 monolayers had greater barrier integrity, likely reflecting the changes that occur following long-term Caco-2 culture including the development of columnar morphology, the formation of microvilli and production of neutral mucus.^46,48,59^ Bacterial translocation does indeed become hindered as Caco-2 monolayers differentiate during long-term culture,^60^ however this model lacks the cell diversity and mucus production observed *in vivo* and thus fails to recapitulate the conditions for bacterial translocation as they are in the human gut.

The most striking difference observed in differentiated colon-derived monolayers was the thick mucus layer. Colonic mucus is composed of an adherent inner mucus layer and a semi-detached outer mucus layer that is continually degraded by microbial proteases.^61^ In humans, the mucus layer is estimated to be as thick as 250–300 µm, and perhaps as thick as 800 µm, and composed primarily of carboxylated (acidic) mucin 2, which is highly interconnected via sulfide linkage creating a gel-like structure.^62,63^ We observed a mucus thickness of 20–40 µm in differentiated colonoid monolayers, reaching approximately 100 µm in semi-wet culture conditions. The thinner mucus layer present in our model may be due to the lack of microbial stimuli, such as lipopolysaccharide and flagellin, that have been shown to promote mucus production *in vivo* .^64^ Nonetheless, a maximum of 10^3^ PFU, representing only 0.01% of viable phage, translocated across differentiated monolayers, suggesting that the thin layer of mucus in our model is capable of capturing phage particles. Accordingly, mucus removal resulted in a 100–1000-fold increase in Ec70 and Kp127 phage translocation, supporting the barrier role of mucus. This protective role is particularly relevant in the context of phage therapy, where orally administered phage preparations come in contact with mucosa in the stomach and small intestine that possess significantly different mucus composition and coverage. While the gastric mucosa possesses similar mucus thickness as the colon, the small bowel contains a significantly thinner and primarily loosely adherent mucus that is easily removed.^63^ These data suggest that orally administered phage may primarily be entering systemic circulation before it reaches the colon, where bacterial targets are concentrated. These data are supported by mouse studies that demonstrate a significant increase in circulating phage as soon as 1 hour post-oral administration.^55^ Due to the loss in phage viability and potential for translocation in the stomach and small bowel, it is not surprising that novel methods such as microencapsulation are being developed to limit phage loss prior to reaching the colon.^65^ Importantly, the use of antacids to improve orally administered phage viability also significantly increases translocated phage titer in blood,^65^ suggesting that alternative methods to target the release of phages to the colon may be safer.

Intestinal permeability and bacterial translocation are elevated in chronic liver and gastrointestinal diseases, so we lastly sought to determine if phage translocation is similarly affected. Interestingly, differentiated colonoid monolayers were more susceptible to alcohol, fat and cytokine induced phage translocation, perhaps due to the significant role of mucus in this context. Ethanol has been shown to increase the thickness of colonic mucus in alcoholics,^66^ and intragastric feeding of alcohol to mice and rats also stimulates an increase in *Muc2* expression, mucus thickness and goblet cell numbers in the ileum and colon.^66,67^ Alcohol may, however, disrupt the barrier function of mucus by dissolving lipids from mucus, leading to a reduction in hydrophobicity and an increase in permeability of the mucus layer.^68^ Fatty acid treatment differentially modulates mucus production, with unsaturated oleic acid promoting mucus production and saturated palmitic acid reducing mucus production and impairing the differentiation of goblet cells.^69,70^ Finally, inflammatory cytokines upregulated in IBD such as IL-1β, IL-6 and TNF-α stimulate multiple gel-forming mucin genes (*MUC2*, *MUC5AC*, *MUC5B*, *MUC6*) *in vitro* .^71^ Secreted mucins were, however, less glycosylated compared to untreated controls; a characteristic that prevents pathogen adherence and mucus rigidity. Mucus thickness and glycosylation varies among different types of IBD with ulcerative colitis (UC) and Crohn’s disease presenting with decreased and increased mucus production/glycosylation, respectively (reviewed in.^72^ Notably, all etiological drivers of intestinal permeability studied herein can modulate tight junction expression and localization, microbial dynamics, and gastrointestinal immune responses, providing multiple mechanisms by which they can stimulate phage translocation in the gut. These factors are particularly important considering we did not define the mechanism by which Ec70 and Kp127 translocation across our monolayer model. In the context of mucus depletion, or reduction in mucus density/adherence in our disease models, it is likely that phage translocation occurs through a combination of transcytosis as described in cell-line monolayer models^13^ as well as intercellular translocation, i.e. through cells and between cells. While we observed translocation occurring within 2 hours in our disease models, it is possible that it may occur even faster, within 10–15 minutes as has been reported previously.^13^ This is ample time for phage translocation in the colon, where colonic transit time ranges from ~ 16–29 h.^73, 74^

Our study found no significant difference in the translocation of Ec70 and Kp127, suggesting that phage morphology and size do not necessarily influence translocation across the colon epithelium. Further studies are required using a variety of gastrointestinal phage species to determine the traits, if any, that facilitate phage translocation through the gut mucosa. It remains possible that size and immunogenicity may dictate the capacity of a phage to move through mucus and epithelial layers in the gut. Exclusion of bacteria in our experiments also limits the conclusions we can draw from this study, as up to 40% of bacterial genomes may express mucolytic enzymes able to degrade colonic mucus and promote phage interaction with the gut epithelium.^20^

In summary, we have generated a colonoid epithelium model that closely mimics the cellular diversity, cell–cell interactions, and mucus content of the human colon. For the first time, this model was used to examine the translocation of phages and revealed that tight junctions and mucus serve as a significant barrier to phage entry in a healthy colonic epithelium. Disruptions to either the cell-cell interactions or mucus content result in enhanced phage translocation. Disease models using the primary insults of ALD, MALFD, and IBD showed increased phage translocation, suggesting that phages may play a role in modulating chronic inflammation in these conditions.

## Supplementary Material

Supplemental Material
