# Supplementary material for: Using a human colonoid-derived monolayer to study bacteriophage translocation
Source: Gut Microbes. 2024 Mar 22;16(1):2331520. doi: 10.1080/19490976.2024.2331520 (PMC10962583; doi:10.1080/19490976.2024.2331520)
Supplement: Supplemental Material [file KGMI_A_2331520_SM8835.docx]

# Supplementary Data

**Table 1. Colonoid culture media**

| Components | Expansion media^41^ | Differentiation media #1^75^ | Differentiation media #2^42^ | Differentiation media #3^43^ |
| --- | --- | --- | --- | --- |
| Advanced DMEM Gibco, #12634010 | 45% | 100% | 100% | 100% |
| L-WRN media ^*^ | 45% | - | - | - |
| Human Wnt3A (Sigma-Aldrich , #H17001) | - | - | - | - |
| Human noggin (Lonza #120-10C) | - | - | 100 ng/ml | 100 ng/ml |
| Human R-spondin 1 (Lonza #120-38) | - | - | 1 µg/ml | - |
| FBS (Gibco, #26140079) | 10% | - | - | 0.1 |
| N2 (Gibco, #17502048) | - | 1x | 1x | - |
| B27 (Gibco, #7504044) | - | 1x | 1x | 1x |
| Glutamax (Gibco, # 35050061) | 2 mM | 2 mM | 2 mM | - |
| EGF (Lonza, #AF-100-15) | - | 50 ng/ml | 50 ng/ml | 50 ng/ml |
| Nicotinamide (Sigma-Aldrich, #329-89-5) | - | - | 10 mM | - |
| Gastrin I (Lonza, #1003377) | - | - | 10 nM | 10 nM |
| N-acetylcysteine (Sigma-Aldrich, #A7250) | - | - | 1 mM | 1 mM |
| HEPES (Sigma-Aldrich, # H4034) | 10 mM | 10 mM | 10 mM | - |
| Gentamicine | 10 µg/ml | - | - | - |
| Pen- Strep (Gibco, #15140122) | 100 U/ml | 100 U/ml | 100 U/ml | 100 U/ml |
| Y-27632 (Cayman, #10005583) | 10 µM | - | 10 µM | 10 µM |
| SB-431542 (Cayman #13031) | 500 nM | - | 500 nM | 500 nM A83-01 |
| SB-202190 (Cayman, # 10010399) | - | - | 10 µM | - |
| DAPT (Cayman, # 13197) | - | - | - | 5 µM |

* L-WRN media was produced from L-WRN cell line (ATCC, #CRL-3276TM) following the manufacturer’s instruction

**Figure S1: Colonoid differentiating markers using 3 different media formulations.** Expression of gene markers for stem cells (*LGR5*) and differentiated intestinal epithelial cells (*MUC2* for goblet cells, *LYZ* for Paneth cells, *CHGA* for endocrine cells and *SI* for enterocytes) were compared among colonoid culture medias and isolated crypts. Mean (red bar) and SEM (black bars) were presented. Ordinary one-way ANOVA test, * p<0.05, ** p<0.01, ** p<0.001.
